# Default mode network deactivation during emotion processing predicts early antidepressant response

**DOI:** 10.1038/tp.2016.265

**Published:** 2017-01-24

**Authors:** M Spies, C Kraus, N Geissberger, B Auer, M Klöbl, M Tik, I-L Stürkat, A Hahn, M Woletz, D M Pfabigan, S Kasper, C Lamm, C Windischberger, R Lanzenberger

**Affiliations:** 1 Department of Psychiatry and Psychotherapy, Medical University of Vienna, Vienna, Austria; 2 MR Center of Excellence, Center for Medical Physics and Biomedical Engineering, Medical University of Vienna, Vienna, Austria; 3 Social, Cognitive and Affective Neuroscience Unit, Department of Basic Psychological Research and Research Methods, Faculty of Psychology, University of Vienna, Vienna, Austria

## Abstract

Several previous functional magnetic resonance imaging (fMRI) studies have demonstrated the predictive value of brain activity during emotion processing for antidepressant response, with a focus on clinical outcome after 6–8 weeks. However, longitudinal studies emphasize the paramount importance of early symptom improvement for the course of disease in major depressive disorder (MDD). We therefore aimed to assess whether neural activity during the emotion discrimination task (EDT) predicts early antidepressant effects, and how these predictive measures relate to more sustained response. Twenty-three MDD patients were investigated once with ultrahigh-field 7T fMRI and the EDT. Following fMRI, patients received Escitalopram in a flexible dose schema and were assessed with the Hamilton Depression Rating Scale (HAMD) before, and after 2 and 4 weeks of treatment. Deactivation of the precuneus and posterior cingulate cortex (PCC) during the EDT predicted change in HAMD scores after 2 weeks of treatment. Baseline EDT activity was not predictive of HAMD change after 4 weeks of treatment. The precuneus and PCC are integral components of the default mode network (DMN). We show that patients who exhibit stronger DMN suppression during emotion processing are more likely to show antidepressant response after 2 weeks. This is, to our knowledge, the first study to show that DMN activity predicts early antidepressant effects. However, DMN deactivation did not predict response at 4 weeks, suggesting that our finding is representative of early, likely treatment-related, yet unspecific symptom improvement. Regardless, early effects may be harnessed for optimization of treatment regimens and patient care.

## Introduction

First-line antidepressant treatment leads to response rates of ~50%, resulting in a large population of patients that does not respond to initial antidepressant treatment.^1^ On the other hand, depression is associated with detrimental personal, social and economic costs. Therefore, neuroimaging biomarker studies have focused on strategies that allow for prediction of treatment response and selection of effective treatment for individual patients in order to optimize clinical routine and patient experience.^2^

In particular, identification of patients that exhibit swift, early response to a particular antidepressant medication may reduce patient suffering, especially as it has been shown that early symptom improvement may predict later course of disease. For example, a meta-analysis of 41 studies showed that symptom reduction at 2 weeks predicted stable response and remission with 81% and 87% sensitivity, respectively.^3^ In fact, such observations have led some authors to suggest shortening the recommended time during which antidepressant effects are assessed and after which antidepressant medication should be adapted, to 2–4 weeks.^4^ As early response serves as an indicator of later outcome, elucidation of early responders before treatment begin may allow for prediction of general treatment results.

Recent studies suggest that data obtained via magnetic resonance imaging (MRI) at baseline predict response to subsequent antidepressant therapy. For example, structural values such as regional gray matter volume^5, 6, 7, 8^ and white matter microstructure^9^ were shown to predict outcome following antidepressant psychopharmacologic treatment. A similar approach has been taken with functional MRI (fMRI). Blood-oxygen-level-dependent (BOLD) response during impulse control,^10^ in response to painful stimuli,^11^ and related to memory encoding^12, 13, 14^ predicted antidepressant response.

In particular, response prediction studies utilizing fMRI have focused on tasks assessing emotion processing. On the one hand, responders and non-responders were shown to differ before initiation of antidepressant treatment. Responders exhibited greater activity in premotor regions, whereas non-responders showed higher insular activity.^15^ Similarly, patients who showed better response to 4 weeks of antidepressant treatment displayed higher activation in the posterior cingulate cortex (PCC), dorsomedial prefrontal cortex and superior frontal gyrus.^16^ Other studies have directly correlated emotion-processing-related fMRI activity assessed before treatment with later antidepressant response. Higher activity in premotor regions, the PCC,^15^ anterior cingulate cortex,^17^ lingual gyrus, hippocampal region, and cerebellum^18^ predicted symptom reduction at 6–8 weeks. Lower ventrolateral prefrontal cortex^19^ and amygdala^20^ activity was related to greater betterment of anhedonia and general depressive symptom improvement, respectively, after 8 weeks of treatment. These results highlight that the regional activation patterns shown to date to have a predictive value are quite heterogeneous. This variability may be a reflection of clinical factors, such as the antidepressant applied.^20^ In fact, it has been shown that fMRI of emotion processing allows differential prediction of response to unique antidepressants. For example, brain activity during emotion processing was shown to differentially predict response to mirtazapine versus venlafaxine.^21^ Furthermore, the time frame after which antidepressant response is assessed may also have a role, highlighting the importance of investigating the predictive value of emotion-processing-related brain activity at different time points, including early response after 2 weeks.

This focus on emotion processing is not surprising, considering the multitude of behavioral,^22^ task fMRI, and functional connectivity^23, 24, 25, 26^ studies demonstrating alterations in major depressive disorder (MDD) and changes over the course of antidepressant treatment.^25, 26, 27, 28, 29, 30^ However, emotion processing is considered a conglomerate of integrated complex processes, and fMRI tasks inevitably focus on only a few aspects. For example, emotion discrimination (EDT) or emotional face-matching tasks are thought to assess voluntary attentional control,^31^ which is understood as a top–down control aspect of emotion processing aimed at focusing attention while preventing distraction from emotionally salient input.^31, 32, 33^ The EDT leads to activation of emotion (prefrontal cortex, orbitofrontal cortex, anterior cingulate cortex, striate and extrastriate cortex, amygdala, hippocampal and parahippocampal) and face-processing regions (fusiform gyri, inferior parietal regions, and frontal eye fields).^34, 35, 36, 37, 38, 39, 40^ In depressed patients, processing of emotional faces is associated with higher activity in the dorsolateral prefrontal cortex, precentral gyrus, anterior cingulate cortex, amygdala, and superior frontal cortex, as well as lower activity in the insula, temporal- and occipital cortices^34, 41, 42^ when compared with controls. Psychopharmacologic antidepressant treatment, on the other hand, is associated with reduction in EDT-related amygdala activation.^43^

In summary, numerous investigations have highlighted the integral role of emotion processing to depression’s pathophysiology, symptoms and treatment. Accordingly, the potential to utilize BOLD during emotion-processing tasks for prediction of antidepressant treatment outcome has been shown. However, the relevance of such patterns for prediction of early improvement of symptoms, after 2 weeks of treatment, is yet to be determined. Early response may be understood as the clinical ‘turning point’ in antidepressant treatment. We therefore aimed to assess whether neural activity related to emotion discrimination may be harnessed for prediction of early antidepressant response, and how predictive measures relate to more sustained antidepressant outcome, using the EDT and 7T fMRI.

## Materials and methods

### Subjects

Twenty-three patients (16 female and 7 male subjects) aged 18–50 years with MDD were included in this study. MDD was diagnosed according to the Diagnostic and Statistical Manual of Mental Disorders (4th edition, text rev.; DSM-IV-TR; American Psychiatric Association, 2000). The Structured Clinical Interview for DSM-IV for Axis I and Axis II disorders (SCID-I and SCID-II for DSM-IV) was utilized to diagnose MDD and exclude the presence of psychiatric Axis I or Axis II comorbidities. Patients had to be free from all medications and may not have taken any psychopharmacologic substance for at least 3 months before inclusion. At screening, all participants underwent standard medical testing including a physical examination, routine laboratory testing and electrocardiography as well as a thorough medical history in order to exclude severe internal or neurological illnesses. In female subjects, urine pregnancy testing was performed to exclude pregnancy, and breastfeeding females were excluded from the study. In all patients, drug-urine tests were performed to exclude current substance abuse. Furthermore, all participants were screened for MRI contraindications including implants, pacemakers or claustrophobia. All subjects provided written informed consent and received financial reimbursement for participation. This study was approved by the Ethics Committee of the Medical University of Vienna and was performed according to the Declaration of Helsinki. Estimation of sample size was based on the assumption of 50% symptom reduction in at least 50% of patients.^1^ For the resulting Cohen's *d* of 0.60, an alpha-error of 0.05 and power of 0.80, 19 or more subjects are required.

### MRI scanning

All subjects underwent 7T fMRI (Siemens Magnetom, Siemens Medical Solutions, Erlangen, Germany) at the Medical University of Vienna using a 32-channel head coil. MRI scanning was well tolerated by participants. fMRI data were acquired using a single-shot gradient-recalled EPI (repetition time (TR)=1.4 s, echo time (TE)=23 ms, matrix size 128 × 128 voxel, field of view (FOV) of 192 × 192 mm, 78 slices of 1 mm with 0.25 mm gap). This study utilized a high-resolution 7T fMRI protocol using multiband (factor 3) imaging with interleaved recording as well as optimized excitation pulses and readout bandwidths. fMRI of amygdala and prefrontal regions is potentially challenging because of susceptibility changes along tissue borders (brain–skull–air) and resulting field inhomogeneities, which may result in signal loss via intravoxel dephasing effects.^44^ However, high-resolution 7T fMRI imaging is thought to improve signal-to-noise ratio and improve sensitivity.^40, 45^

### Emotion discrimination task

A version of the EDT, a block-design task in which patients are instructed to recognize visually presented basic emotional facial expressions, was used as previously published in Windischberger *et al.*^43^ and based on Hariri *et al.*^35, 37^ The task was presented using Cogent toolbox for MATLAB. In the test condition (EDT), two faces exhibiting different facial expressions were depicted at the bottom of the screen while a third emotional face was presented at the top of the screen. Patients were instructed to pair the top face with the bottom face that was most similar in emotional expression. In the control condition, the object discrimination task (ODT), patients were instructed to match shapes presented in the same position as the faces in the test condition on backgrounds of similar color distribution. The patients denoted their choice via button press. The task was performed over the course of 9 min, including four alternating blocks each of the EDT and ODT conditions, starting with the EDT condition. Each picture was presented for at least 2 s and patients had up to 5 s in order to submit the button press.

### Preprocessing

fMRI data preprocessing comprised slice-timing correction, realignment, spatial normalization and spatial smoothing as implemented in SPM12 (Wellcome Trust Centre for Neuroimaging, http://www.fil.ion.ucl.ac.uk/spm) using default parameters, unless otherwise specified. Slice-timing correction, using the slices recorded at TR/2 as reference,^46^ and motion correction, using the mean image as reference, were performed. In order to further limit motion artifacts, each patient’s head was fixated in the head rest using soft foam cushions to minimize movement during scanning. Framewise displacement (FD), a measure of in-scanner motion, was calculated.^47^ For our patient group, the mean±s.d. FD=0.23±0.13 mm. The generally accepted maximum FD=0.5 mm.^48^

### Medication

Following the MRI scan, all patients were treated with Escitalopram according to a flexible dose antidepressant treatment protocol based on the recommended dosing starting with 10 mg per day.^49^ Escitalopram is considered a first-line antidepressant treatment.^49, 50^ In patients with pronounced agitation and anxiety symptoms, treatment was phased in with 5 mg per day in order to improve tolerability. In these patients, treatment was subsequently increased to 10 mg per day within the first week of treatment, with the exception of one patient who was kept on 5 mg. Further dose adaptation took place after 2 weeks of Escitalopram treatment in patients in whom HAMD did not decrease by at least 50%. At this time point, the dose was increased by 5 or 10 mg, depending on side effects and tolerability, based on clinical inspection of patients. In order to ensure adherence to treatment, it was announced that plasma levels would be assessed over the course of the study.

### Psychological tests

The 24-item HAMD, performed by an experienced psychiatrist, was used to assess depressive symptoms at the MRI visit, and after 2 (mean±s.d.=13.83±5.13 days) and 4 (27.64±4.66 days) weeks of Escitalopram treatment. HAMD at 2 weeks was utilized to assess early effects of antidepressant treatment. HAMD was also measured at 4 weeks in order to investigate whether a possible predictive value of fMRI data at 2 weeks carried over to prediction of more sustained antidepressant response. Early symptom improvement was defined as a reduction in HAMD scores by at least 20% after 2 weeks of treatment based on Szegedi *et al.*^3^ Response was defined as reduction in the HAMD score by at least 50% in comparison with baseline. Remission was defined as ⩽7 points on the HAMD scale.

### Statistical analyses

Statistical analysis of fMRI data was performed with SPM12. First-level analysis for elucidation of task-specific activation consisted of the contrast test (EDT) versus control (ODT) condition as well as EDT versus baseline brain activity. Voxel-wise regression analysis was performed between task-specific neural activity (contrast EDT versus ODT) and HAMD reduction after 2 as well as 4 weeks of Escitalopram treatment. Age, sex and baseline symptom severity, defined as HAMD on the day of MRI, were included as covariates. Second-level analyses were corrected for multiple testing using Gaussian random field theory as implemented in SPM12 and the threshold for significance was set at *P*⩽0.05 family-wise error (FWE)-corrected at the cluster-level following *P*⩽0.001 uncorrected at the voxel-level. Variation of data is covered in *t*-test and regression analyses by definition via the influence of s.d. SPM12 deals with characteristics of fMRI data violating necessary statistical assumptions (such as autocorrelation) during processing on the subject level.

## Results

### Clinical data

The 23 investigated MDD patients showed the mean symptom severity at baseline HAMD±s.d.=26.26±5.64. One patient was subsequently excluded from fMRI analysis by visual inspection due to excessive motion artifacts. One patient was lost to follow up at 4 weeks. At 2 weeks, early improvement, response and remission rates were 10/22 (45.45%), 4/22 (18.18%), and 1/22 (4.55%). By 4 weeks, 4/21 (19.05%), and 5/21 (23.81%) were in response and remission, respectively.

### Task-related activity

The EDT revealed brain activity patterns in accordance with those described in the literature.^34, 35, 36, 37, 38, 39, 40^ The contrast EDT versus ODT resulted in activation of the superior and inferior frontal cortex and the superior and middle temporal cortex, as well as amygdala, hippocampus, parahippocampus, calcarine, lingual, fusiform, and cerebellar regions. Task-related deactivation was found in the middle and inferior occipital cortex, as well as fusiform and cerebellar regions.

**Figure 1:**
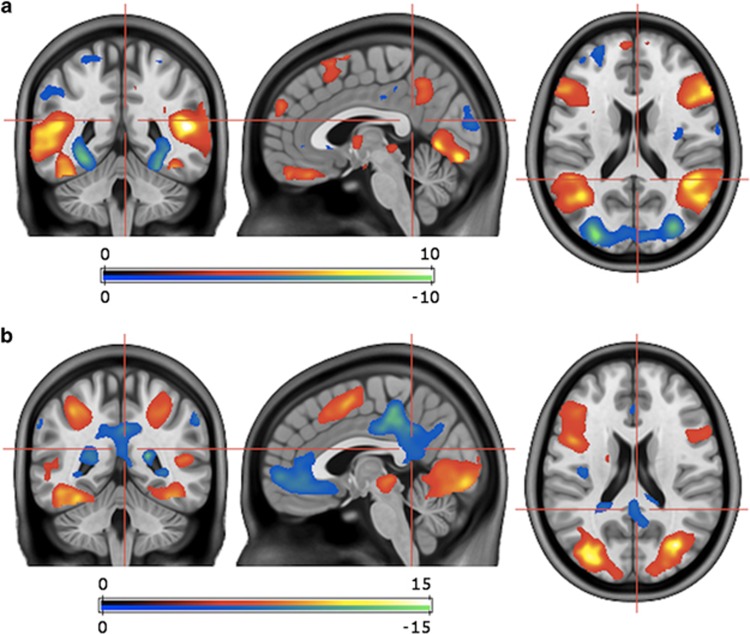
Emotion-processing-related brain activity assessed with the EDT and 7T fMRI. Figure exhibits contrast of EDT versus ODT (**a**) and EDT versus baseline (**b**) BOLD response, which was used to elucidate emotion-processing-related brain activity. Performance of the EDT results in activation of emotion- and face-processing regions^34, 35, 36, 37^ and deactivation of regions involved in the DMN,^52^ as was the case in our group of MDD patients (Table 1). Performance of the EDT specifically elucidates attentional control as a top–down control aspect of emotion processing.^31, 32, 33^ T-threshold for *P*⩽0.001 uncorrected, voxel-level=3.53 (**a**, **b**); *P*⩽0.05 FWE-corrected, cluster-level *k*=443 (**a**), 57639 (**b**). BOLD, blood-oxygen-level-dependent; DMN, default mode network; EDT, emotion discrimination task (test condition); fMRI, functional magnetic resonance imaging; FWE, family-wise error; ODT, object discrimination task (control condition); MDD, major depressive disorder.

Contrasting EDT versus baseline task activity resulted in activation of the superior, middle, and inferior occipital cortex, the superior and inferior temporal cortex, the superior and inferior parietal cortex, the gyrus angularis, and the middle frontal cortex. Furthermore, this cluster included activation of lingual, calcarine, fusiform, and cerebellar regions. For the contrast EDT versus baseline, deactivation was found in the anterior, middle and PCC, the precuneus, the middle frontal cortex, and parietal regions, including the gyrus angularis and inferior parietal cortex (Figure 1).

All activities reported were significant at *P*<0.05 FWE-corrected at the cluster-level; for peak statistics and coordinates see Table 1. Task accuracy was 100%. In all stimulus sets, all patients correctly matched the presented faces based on the emotions they expressed.

### Regression analysis

**Figure 2:**
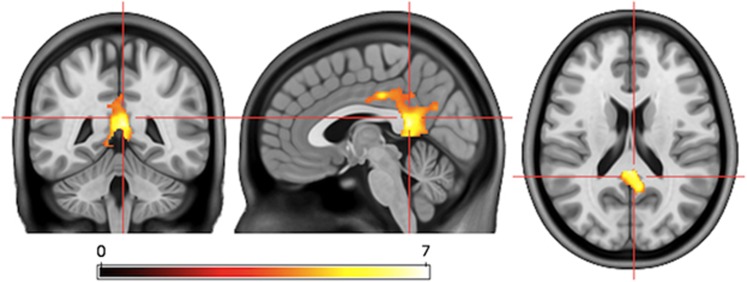
Deactivation in the precuneus and PCC correlates with early response. Regression analysis revealed a cluster of deactivation with peak in the precuneus and stretching to the PCC isolated using the contrast EDT versus ODT that correlated positively (*P*<0.001, FWE-corrected, cluster-level) with antidepressant response assessed as HAMD reduction after 2 weeks of treatment. The precuneus and PCC are considered central components of the DMN.^51^ Therefore, across our group of MDD patients, the stronger the deactivation of the DMN during emotion processing was at baseline, the more HAMD decreased after 2 weeks of Escitalopram treatment. T-threshold for *P*⩽0.001 uncorrected, voxel-level=3.65; *P*⩽0.05 FWE-corrected, cluster-level *k*=233. DMN, default mode network; EDT, emotion discrimination task (test condition); FWE, family-wise error; HAMD, Hamilton Depression Rating Scale; MDD, major depressive disorder; ODT, object discrimination task (control condition); PCC, posterior cingulate cortex.

**Figure 3:**
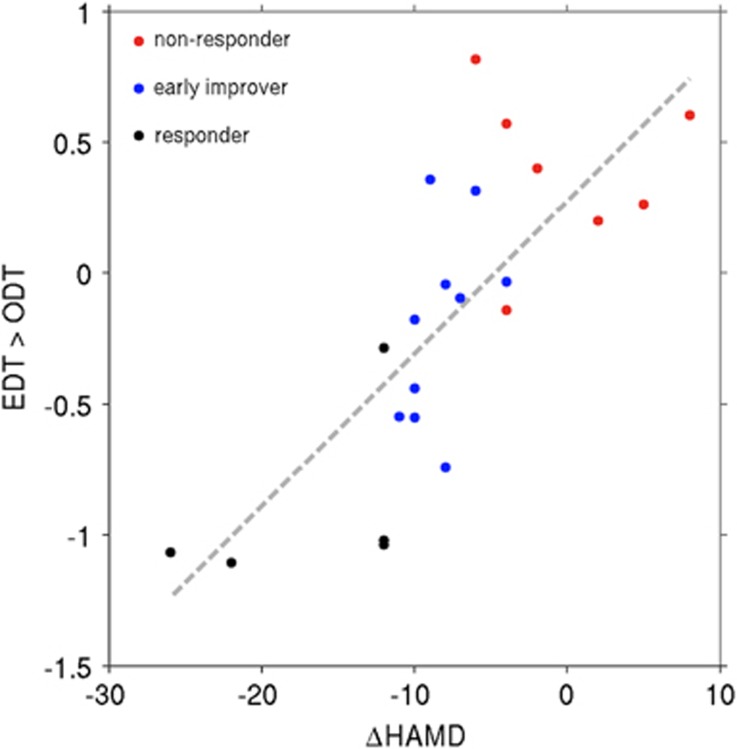
Deactivation of the DMN during emotion processing predicts early antidepressant response in MDD. Peak deactivation of a cluster within the precuneus and PCC (contrast EDT versus ODT, *y* axis) before start of antidepressant treatment predicted subsequent antidepressant response (absolute HAMD change, *x* axis) in regression analysis. The color spots denote individual patients with early symptom improvement (blue)=HAMD reduction⩾20%, early response (black)=HAMD reduction⩾50% and non-response (red)=HAMD reduction <20%, all after 2 weeks of Escitalopram treatment. Patients are dispersed along the line of regression with non-responders showing the least deactivation and responders the most deactivation of the precuneus and PCC, regions known to be part of the DMN.^51^ DMN, default mode network; EDT, emotion discrimination task (test condition); HAMD, Hamilton Depression Rating Scale; MDD, major depressive disorder; ODT, object discrimination task (control condition); PCC, posterior cingulate cortex.

For EDT versus ODT, deactivation in the precuneus and the PCC correlated with early antidepressant response defined as change in HAMD scores between baseline and 2 weeks after treatment begin (Figures 2 and 3). This correlation was significant after correction for symptom severity at baseline assessed with HAMD, age, and sex (*P*<0.001, FWE-corrected, cluster-level). In order to further correct for a possible influence of disease severity, we investigated whether baseline HAMD correlated with EDT versus ODT brain activity, which it did not.

The cluster that correlated with early response overlapped with baseline task activity. More specifically, this region exhibited deactivation during the contrast EDT versus baseline (*P*<0.05, FWE-corrected, cluster-level) as well as for the contrast EDT versus ODT, yet below the significance threshold. In addition, clusters within the left middle temporal cortex, left hippocampus and right gyrus rectus isolated in regression analysis also overlapped with EDT versus baseline task activity. Interestingly, no correlation was found in brain wide-analysis of baseline EDT versus ODT activity with HAMD change after 4 weeks of Escitalopram treatment.

In order to investigate the findings for a potential bias due to outliers, delete-1 jackknife resampling procedure was performed. This analysis indicated little bias of the associations for correlations of the peak voxel (observed *r*=0.858, corrected *r*=0.855) and the entire cluster (observed *r*=0.832, corrected *r*=0.833).

All activities reported were significant at *P*<0.05 FWE-corrected at the cluster-level; for peak statistics and coordinates see Table 2.

## Discussion

The study at hand aimed to investigate whether fMRI task activity related to emotion processing allows for prediction of early antidepressant response. We found that deactivation of a cluster in the precuneus and PCC (EDT versus ODT) predicted HAMD reduction after 2 weeks of treatment. Interestingly, PCC and precuneus deactivation did not correlate with symptom improvement after 4 weeks of treatment.

The PCC and precuneus are considered integral components of the default mode network (DMN).^51^ A central characteristic of the DMN is its deactivation during performance of demanding cognitive or emotional tasks.^52^ Deactivation of the DMN increases with the level of attention a task requires^53^ and with successful performance,^54^ likely as a result of reallocation of resources. Appropriately, in our study, the precuneus and PCC were also deactivated during performance of the EDT, which is designed specifically to elucidate attentional control of emotional stimuli.^31^ Other regions also typically implicated in the DMN, including the angular gyrus and inferior parietal regions, inferior frontal regions, and middle frontal regions,^55^ also showed deactivation during emotion processing in our study. Therefore, the presumed attentional control exerted during the EDT resulted in DMN suppression in our group of MDD patients, with stronger deactivation of the DMN predicting better subsequent response to 2 weeks of antidepressant treatment.

The DMN is typically related to self-referential processes^56^ and active during mind-wandering states.^57^ The precuneus and PCC have been considered central moderators within the DMN in that they are highly connected to other regions of the network.^58^ Furthermore, the precuneus connects the DMN to cognitive control networks and has been implicated in the regulation of cognitive states.^59, 60, 61^ The DMN can be divided into functional subsets, described as the medial–temporal and dorsal–medial system by some authors,^62^ which overlap broadly with the anterior and posterior subsets defined by other authors.^63^ In fact, the PCC is addressed by the first of these models as a core component of the DMN, which coordinates communication between regions responsible for functions such retrieval of episodic information relevant to salient input, and others responsible for mentalization. Activity within the PCC has been shown to correlate with activity within all other regions of the network, and it is among the most consistently enlisted regions in the DMN.^62^ The precuneus and PCC are often addressed in the literature as part of a common functional cluster in the DMN,^51^ reflecting the pattern we found.

Changes to the activation of—and the connectivity between—components of the DMN have repeatedly been shown in depression and antidepressant treatment.^64, 65^ Numerous studies have demonstrated insufficient task-related deactivation of the DMN in depression.^66, 67, 68, 69^ Deficient deactivation of the DMN may result in interference from DMN-processed information during cognitive or emotional tasks, which may be accompanied by symptoms such as rumination^66^ or cognitive deficits.^67, 70, 71^ Furthermore, depressed patients demonstrate functional dissociation between subcomponents of the network. For example, altered interplay between its functional subsets has been shown,^63, 72^ and depressed patients show subset-specific changes to functional connectivity within the DMN. In fact, functional connectivity within the DMN may correlate with depressive symptoms in some subsets, while not, or even to a negative extent, in others.^63^ It is therefore fitting that the deactivation we found to correlate with early antidepressant response is located in the PCC and the precuneus, and does not stretch across all regions of the network.

Numerous previous fMRI studies have demonstrated that unique subsets of depressed patients demonstrate differential brain activation during emotion processing. For example, neural activity during emotion regulation may be reflective of certain trait characteristics relevant to depressive disorders. Along this line, personality traits such as extroversion, reward drive, tendency towards aggression,^73^ propensity for suppression of emotions,^74^ neuroticism,^75^ and trait anxiety^76^ are reflected in the brain activity measured in fMRI emotion-processing tasks. On the other hand, comorbidities commonly demonstrated by depressed patients, in particular anxiety, are also reflected in neural activation related to emotion processing.^77^ These findings underscore the concept that differential brain activity may be descriptive of certain patient subgroups within the diagnosis of MDD. These activity patterns, as well as the behavioral, cognitive or emotional aspects with which they are associated, may in turn also be understood as a ‘signature,’ which is reflective of propensity for a certain course of disease. Along this line, our results suggest that the extent of deactivation of the DMN during emotion processing may be descriptive of the extent to which patients show advantageous response to 2, although not 4 weeks of antidepressant treatment.

We propose that DMN suppression may actually predict early unspecific treatment factors, such as placebo response or anticipation of symptom improvement, which likely do not extend to 4 weeks. Both the predictive value of DMN suppression at 2, but not at 4 weeks, as well as the finding that our patients showed high rates of at least early improvement (15/22, 68.18%) at 2 weeks,^3^ a trend that did not carry over to response or remission at 4 weeks, support this concept. Furthermore, in our patient group, patients who responded at 2 weeks were not necessarily those who responded to 4 weeks of treatment. Of the 10 patients who showed early improvement (⩾ 20% HAMD reduction) at 2 weeks, 3 showed response (⩾ 50% HAMD reduction) and 1 was in remission (⩽7 HAMD points). Of the four patients who showed response at 2 weeks, response persisted in one patient and three were in remission. Of the eight patients who did not show at least early improvement at 2 weeks, one patient responded at 4 weeks. Therefore, although this question cannot be meaningfully statistically analyzed in a subject group of this size, our results numerically reflect this assumption.

Accordingly, resting state functional connectivity of the salience network was shown to predict response to 1 week of placebo treatment in an antidepressant trial.^78^ Considering the fundamental role of the salience network in emotion processing,^79^ this finding suggests that a patient’s neurobiological correlates of emotion processing may predict unspecific treatment effects. Our finding of response prediction by emotion-processing-related DMN suppression at 2, yet not at 4 weeks underlines this concept.

The possible relevance of a placebo effect again supports that the extent of DMN deactivation may be reflective of certain MDD patient subgroups. Based on our results, DMN suppression is indicative of patients that may be more likely to exhibit early, possibly unspecific, clinical responses. This concept is emphasized by literature, which highlights that insufficient DMN suppression in depressed patients may be reflective of depressive subtypes characterized by certain symptoms.^66, 67, 70, 71^ Based on our study, patients who show less symptom improvement to 2 weeks of treatment, including unspecific treatment effects, may be lacking in what can be understood as a physiologic neurobiological resource that fosters effective emotion processing, likely by suppressing interfering self-referential information.^66, 80^

Molecular imaging studies further emphasize that the observed relationship between DMN suppression and symptom improvement at 2 weeks may be a reflection of early, unspecific treatment effects. Antidepressant treatment regimes have long been based on response times of at least 4 weeks and the increasing emphasis on early evaluation of response and adaptation of treatment strategy has only recently come to light.^4^ Molecular imaging literature emphasizes that secondary regulatory mechanisms within the serotonergic system, which likely occur with a latency of several weeks, are in fact the true mediators of antidepressant response to selective serotonin reuptake inhibitors such as Escitalopram.^81, 82, 83^ One may therefore question whether 2 weeks are sufficient for these processes to take place and induce clinical antidepressant effects.

One may postulate that severity of depressive symptoms, which of course influences the clinical course of a depressive episode, may be reflected in EDT brain activity, hereby influencing the results of our regression analysis. However, the correlation between deactivation of the precuneus/PCC cluster during emotion processing and symptom improvement remained significant after correction for baseline symptom severity, as well as age and sex. The lack of an influence of symptom severity is further underscored by the observation that baseline HAMD did not correlate with EDT versus ODT brain activity. The EDT was chosen for the elucidation of neural activity related to regulation of emotion.^31, 32, 33^ The EDT activity we observed was highly reflective of the brain activation patterns typically published for this task,^34, 35, 36, 37, 38, 39, 40^ therefore demonstrating that the EDT was successfully employed in our study (Table 1 and Figure 1).

Early treatment effects, whether they are unspecific or not, can be considered a relief for patients with MDD at the beginning of the treatment process. These effects can be harnessed to improve treatment adherence and patient motivation. Along this line, recent developments in antidepressant psychopharmacology have focused on short-term treatment strategies such as ketamine.^84^ However, from a clinical stand point our results emphasize that patients with insufficient DMN suppression, which predicts 2, yet not 4 week response, should be followed up with regularly early in treatment.

The small sample size of our study, although typical of neuroimaging studies, must be discussed as a limitation. Owing to the high clinical and neurobiological variability within depression as a disease cluster,^85^ clinically viable prediction measures will require studies with extensive sample sizes to allow for differentiation of unique patient subgroups. However, although the smaller sample size of our study can be discussed as a limitation, studies such as ours are essential for understanding the neural mechanisms that underlie antidepressant effects, a prerequisite for large-scale prediction studies. In addition, future studies should relate our findings to even more long-term treatment outcome, in particular as our results speak for a possible time point-specific predictive potential of fMRI activity.^85^ The aim of the current study is, generally speaking, to establish the relationship between fMRI findings and clinical correlates, using a whole-brain approach to limit bias. Therefore, our study benefits from the use of 7T MRI, which is associated with high signal-to-noise ratio and sensitivity.^40, 45^ However, considering that 7T is not yet broadly available in clinical practice, results should be confirmed in future investigations using 3T imaging.

In summary, we find that DMN deactivation during an emotion-processing task predicts early response to Escitalopram treatment. Response prediction at 2, yet not at 4 weeks of treatment suggests that DMN suppression during emotion processing may be predictive of early, possibly unspecific, treatment effects. This concept is underlined by studies demonstrating the relevance of emotion regulation’s neural correlates to placebo response,^78^ evidence on the serotonergic molecular processes mediating response to selective serotonin reuptake inhibitors,^81, 82, 83^ and studies highlighting that DMN suppression is likely reflective of specific depressed patient groups.^66, 67, 70, 71^ A wealth of literature links deficient suppression of the DMN to depression.^86, 87, 88^ This is, however, to our knowledge the first study to demonstrate a relationship between DMN suppression assessed with fMRI and early treatment effects. From a clinical stand point, early symptom improvement, whether persistent or not, can be harnessed for optimization of treatment regimens and patient care.

## Figures and Tables

**Table 2:** Regression analysis EDT versus ODT task activity and HAMD reduction at 2 weeks

*Regions*	*MNI coordinates, peak, mm*			*Cluster statistics*	
	x	y	z	*Cluster size*	T
*HAMD baseline, age, and sex as covariates*					
Gyrus rectus, R	8	28	−18	109	7.48*
Middle temporal cortex, L	−66	−18	−18	421^##^	6.83
Precuneus, L	−4	−46	14	1431^###^	6.67
Hippocampus, L	−18	−24	−6	308^#^	5.58
Middle temporal cortex, R	68	−34	−6	233^#^	5.18

Abbreviations: AAL, Automated Anatomical Labeling Atlas; EDT, emotion discrimination task; FWE, family-wise error; HAMD, Hamilton Depression Rating Scale; ODT, object discrimination task; L, left; R, right.

Regions according to AAL.

FWE-corrected, voxel-level:

*P<0.05.

FWE-corrected, cluster-level (voxel size 2 × 2 × 2 mm):

^###^*P*<0.001; ^##^*P*<0.01; ^#^*P*<0.05.

**Table 1:** Emotion discrimination task brain activity

*Regions*	*MNI coordinates, peak, mm*			*Cluster statistics*	
	x	y	z	*Cluster size*	T
*EDT versus ODT*					
Middle temporal cortex, L	−56	−62	12	4036	11.63***
Amygdala, L	−24	−4	−18		9.27***
Inferior frontal cortex, pars triangularis, L	−56	30	0	3777	9.77***
Middle temporal cortex, R	50	−64	10	3221	9.64***
Superior temporal cortex, R	48	−44	14		8.29**
Hippocampus, R	20	−8	−16	707	9.14**
Amygdala, R	28	0	−22		8.17**
Calcarine region, L	−4	−82	−12	1117	8.54**
Fusiform region, R	42	−52	−22	930	7.70**
Cerebellum, R	38	−44	−24		7.12*
Fusiform, L	−42	−50	−18	95	7.52**
Inferior frontal cortex, pars triangularis, R	40	20	24	1892	6.96*
Precuneus, L/R	0	−54	36	556	6.88*
Superior frontal cortex, pars orbitalis, L	−8	38	−24	443^##^	6.38
					
*ODT versus EDT*					
Middle occipital cortex, R	28	−90	16	1570	11.26***
Superior occipital cortex, R	22	−60	36	523^##^	5.56
Middle occipital cortex, L	−32	−82	14	1479	11.16***
Inferior occipital cortex, L	−34	−78	−2		6.92***
Cerebellum, L	−28	−54	−16	763	9.92***
Fusiform, R	30	−60	−10	789	9.59***
Superior temporal cortex, R	50	−4	−6	401^##^	6.35
Superior parietal cortex, L	−20	−62	44	613^##^	6.23
Inferior parietal cortex, L	−50	−28	40	276^#^	5.55
					
*EDT versus baseline*					
Inferior occipital cortex, R	48	−74	−2	57 639	23.31***
Middle occipital cortex, L	−32	−88	8		21.41***
Middle frontal cortex, pars orbitalis, R	20	42	−18	161	7.06*
					
*Baseline versus EDT*					
Middle cingulate cortex, L/R	0	−30	46	9906	17.51***
Gyrus angularis, R	58	−60	38	665	15.75***
Gyrus angularis, L	−46	−66	42	1148	12.29***
Inferior parietal cortex, L	−58	−50	40		10.45***
Middle frontal cortex, pars orbitalis, L	−6	56	−2	4439	9.88***
Anterior cingulate cortex, L	−2	38	−6		8.79**
Superior temporal cortex, L	−44	−18	0	150	7.69**
Parahippocampal gyrus, L	−20	−20	−24	429^##^	6.18
Middle frontal cortex, L	−22	34	42	867^###^	6.16
Middle temporal cortex, L	−58	−18	−16	802^###^	5.92

Abbreviations: AAL, Automated Anatomical Labeling Atlas; EDT, emotion discrimination task; FWE, family-wise error; ODT, object discrimination task; L, left; R, right.

Regions according to AAL.

FWE-corrected, voxel-level:

***P<0.001; **P<0.01; *P<0.05.

FWE-corrected, cluster-level (voxel size 2 × 2 × 2 mm):

^###^*P*<0.001; ^##^*P*<0.01; ^#^*P*<0.05.
